# To Cheat or Not To Cheat: *Tryptophan Hydroxylase 2* SNP Variants Contribute to Dishonest Behavior

**DOI:** 10.3389/fnbeh.2016.00082

**Published:** 2016-05-02

**Authors:** Qiang Shen, Meijun Teo, Eyal Winter, Einav Hart, Soo H. Chew, Richard P. Ebstein

**Affiliations:** ^1^College of Economics and Management, Zhejiang University of TechnologyHangzhou, China; ^2^Department of Management Science and Engineering, School of Management, Zhejiang UniversityHangzhou, China; ^3^Department of Psychology, Faculty of Arts and Social Sciences, National University of SingaporeSingapore, Singapore; ^4^Center for the Study of Rationality, Hebrew UniversityJerusalem, Israel; ^5^Department of Economics, Hebrew UniversityJerusalem, Israel; ^6^Philosophy, Politics and Economics Program, University of PennsylvaniaPhiladelphia, PA, USA; ^7^Department of Economics, Faculty of Arts and Social Sciences, National University of SingaporeSingapore, Singapore; ^8^Lab for Behavioral Biological × Economics and the Social Science, National University of SingaporeSingapore, Singapore

**Keywords:** neurogenetics, lying, serotonin, TPH2, single-nucleotide polymorphism

## Abstract

Although, lying (bear false witness) is explicitly prohibited in the Decalogue and a focus of interest in philosophy and theology, more recently the behavioral and neural mechanisms of deception are gaining increasing attention from diverse fields especially economics, psychology, and neuroscience. Despite the considerable role of heredity in explaining individual differences in deceptive behavior, few studies have investigated which specific genes contribute to the heterogeneity of lying behavior across individuals. Also, little is known concerning which specific neurotransmitter pathways underlie deception. Toward addressing these two key questions, we implemented a neurogenetic strategy and modeled deception by an incentivized die-under-cup task in a laboratory setting. The results of this exploratory study provide provisional evidence that SNP variants across the *tryptophan hydroxylase 2 (TPH2)* gene, that encodes the rate-limiting enzyme in the biosynthesis of brain serotonin, contribute to individual differences in deceptive behavior.

## Introduction

Men are liars. We’ll lie about lying if we have to. I’m an algebra liar. I figure two good lies make a positive.

Tim Allen

‘Lying,’ as a facet of human nature, has been a focus of research across a broad range of disciplines including psychology, evolutionary biology, and experimental economics. Due to its immoral nature, lying is widely condemned across cultures. However, in addition to its dark side, lying is ubiquitous and also appears essential for frictionless social interactions (Nyberg, 1993; DePaulo et al., 1996).

Lying is generally considered as unethical and immoral behavior and most individuals regard themselves as honest. Nevertheless, it is clearly tempting to deceive when the pay off is sufficiently attractive. Toward understanding how individuals resolve the conflict between self-interest and maintenance of a positive sense of one’s own worth, a growing number of studies implement laboratory-controlled experiments. Mazar et al. (2008) found, not surprisingly, that subjects act dishonestly to benefit themselves but set a limit to their level of dishonesty viz., “A little bit of dishonesty gives a taste of profit without spoiling a positive self-view.”

Notably, the integrity of cognitive function seems to be vital for individuals to refrain for lying. Following a task that requires the exertion of mental self-control, subjects are more likely to deceive suggesting that the depletion of one’s cognitive resources leads to lowering of our moral guard. This explanation is strengthened by studies showing that subjects experiencing ego-depletion due to the wear and tear of daily experiences (Kouchaki and Smith, 2013) or sleep deprivation (Barnes C.M. et al., 2011) are more likely to lie. Recruitment of the dorsolateral prefrontal cortex (DLPFC) is observed when subjects attempt to refrain from misreporting their actual performance in a coin toss paradigm, to financially benefit themselves (Greene and Paxton, 2009). Individuals who behaved dishonestly showed enhanced activity in control-related regions of prefrontal cortex either when behaving dishonestly or when they refrained from dishonesty. These and other (Abe et al., 2007, 2014; Parkinson et al., 2011) neural imaging results strengthen the notion that cognitive control is intricately connected to decisions to lie or not to lie.

Similar to most human traits (Polderman et al., 2015), individual differences in lying behavior show moderate heritability (Ahern et al., 1982; Bond and Robinson, 1988) administered a battery of self-report inventories that measure a total of 54 personality traits to 1819 Hawaiian family members. Familial resemblance was found for many personality characteristics but the strongest and most surprising family similarity was in the tendency to lie, as measured by the Lie Scale of the Eysenck Personality Questionnaire (EPQ). A partial genetic model of lying was further supported by a study that analyzed the EPQ Lie scale responses given by a sample of 543 adult British twin pairs. Lie scores were found to be more similar for monozygotic twins than Dizygotic Twins and a heritability estimate of 0.48 was inferred using structural equation modeling techniques (Young et al., 1980). Similarly, Eaves et al. (1999) in a large study of adult twins and family members reported broad heritability estimates for the Lie scale of 29–42%. More recently, a twin study with a sample size of over 1000 same-sex pairs suggests that genetic component can account for 26% of subjects’ views on avoiding taxes payment and 42% of their personal views on claiming sick benefit while being healthy (Loewen et al., 2013).

Despite the considerable heritability of lying behavior, few if any studies have examined the role of specific polymorphisms in contributing to lying and deception. The current investigation addresses two neglected issues in understanding the biological roots of lying in humans. We use a neurogenetic strategy to identify a neurotransmitter system that mediates lying and deception, and pinpoint specific polymorphisms contributing to individual differences in dishonesty. In modeling the lying phenotype, we focus on a single facet of lying, personal advantage (material gain). We model material gain in the laboratory using the die-under-cup paradigm introduced by Fischbacher and Heusi (2013) that allowed inferences to be drawn about the distribution of cheating in the population. In this task, subjects are asked to report the results of a private six-sided die roll and receive real-money payoffs proportionately to their reported die outcome.

Spence et al. (2004) suggest that deception may be viewed from a cognitive neurobiological perspective as an exercise in behavioral control making use of limited cognitive resources. Such a notion is underscored by studies of the neural underpinnings of deceptive behavior (Greene and Paxton, 2009) discussed above. Interestingly, serotonin neural pathways play a key role in behavioral inhibition and executive function (Barnes J.J. et al., 2011) and disturbances in serotonin metabolism have been consistently reported for subjects with autism, for whom deceptive communication and lying is especially difficult (Cook and Leventhal, 1996). Walther and Bader (2003) identified a gene expressed in the brain stem that encodes the rate-limiting enzyme in the synthesis of 5-HT in humans, mice and rats, tryptophan hydroxylase-2 (*TPH2*). As the rate-limiting enzyme for the synthesis of central 5-HT, *TPH2* plays a key role in the modulation of 5-HT neurotransmission. Numerous association studies have linked *TPH2* genetic variants to a wide spectrum of endophenotypes, behavioral traits and neuropsychiatric diseases (Chen and Miller, 2012). In particular, the association of *TPH2* with attention deficit hyperactivity disorder (ADHD; Manor et al., 2008) is especially relevant to the current study considering the reported deficits in executive control characterized by impulsivity in that disorder. Hence, we hypothesize that SNP variants in the *TPH2* gene will contribute to honest versus dishonest behavior in the die-under-cup laboratory model.

## Materials and Methods

### Subjects

Two hundred and five undergraduates aged between 19 and 30 years (mean = 22 years, *SD* = 1.48 years) were recruited from National University of Singapore (NUS) using Online Recruitment System for Economic Experiments (ORSEE, Ben Greiner^1^). They were randomly selected from samples of an ongoing project of economic decision-making in Singapore and China (B2ESS^2^). Blood samples from 1127 ethinically Han Chinese students from NUS in Singapore from the first wave and saliva samples from 936 Han Chinese students for the second wave have been collected in advance. This study was approved by Institutional Review Board of National University of Singapore and written informed consent forms were obtained from all attending subjects according to the Declaration of Helsinki. A subsample of subjects (*N* = 205) participated in the die-under-cup paradigm at a later date – about 2 years after the initial recruitment and collection of DNA and genotyping. This group solely consisted of Singaporean Han Chinese due to logistic considerations.

### Materials and Design

The die-under-cup paradigm was adapted from Fischbacher and Heusi (2013) in which people roll a die in private and are paid according to the number (i.e., die outcome) they reported. In our study, on arrival, subjects were given a cup with a hole, die and booklet containing two sheets of paper. Following behavioral economics practice, all subjects received monetary remuneration in private (in a separate room) for their participation. Privacy was important so that subjects payment were not observed by other subjects in the study.

This study was a between-subjects design. The independent variables were the *TPH2* genotypes (2 homozygous and 1 heterozygous allele) with gender and age as covariates. The dependent variable was the reported number of the rolled die. Although, it is impossible for us to tell who actually told a lie, we could determine lying on the group level by comparing the observed fraction of reported die-roll outcome with the expected 1/6 probability from a fair die. Lying degree was measured by the discrepant reported mean die-roll outcome at the group level. It is assumed that the higher the mean die-roll outcome reported, the higher the tendency that the examined group cheated and vice versa.

### Experimental Procedure

Subjects arrived at the lab in groups varying from 8 to 19 in number. As the die-under-cup task was relatively short and in order to make it immune to potential confounds from other experimental tasks, it was carried out as the first task in a three-experiment session. To prevent the subjects from guessing the true purpose of the experiment, they were informed that the task was added to determine an additional payoff as a token of appreciation for their participation in the other two main experiments. Subjects were spaced such that there was a vacant space on either side of each subject; they could not see the dice outcomes reported by others. This is to prevent the infectiousness of cheating behavior mentioned by Gino et al. (2009) wherein cheating can be increased by observing the bad behavior of others around (e.g., a subject who observe someone reporting a “6” might just follow suit regardless of the individual’s own internal moral compass). Subjects were not debriefed following their participation in the study to avoid possible feelings of discomfort since the experiment was focused on dishonest behavior. Subjects were simply paid according to their reported die throw. Anonymity was maintained following standard IRB protocols involving genetic material. All DNA is coded and students are identified by their DNA codes with no use of personal names. Laboratory workers, etc. have no access to any personal information except on a need to know basis following consultation with the PI. The DNA code linking to personal information is on a locked computer in the PI’s personal office at the University.

### Die-Under-Cup Paradigm

The procedures of the die-under-cup paradigm were closely adapted from Fischbacher and Heusi (2013). Subjects were instructed to roll a six-face die under a cup once, check the outcome, memorize it and then roll two more times to make sure that the die was fair. This procedure allowed the subjects to hide their first roll even after they left the experiment. This feature – ensuring total anonymity – made it impossible to detect lying on the individual level, yet ensure that our data depict the overall subjects’ real propensity to lie. The experimenter demonstrated the procedures before subjects began the actual task. Subjects were told that the reported outcome will correspond to the amount of payoff they would receive at the end of the experiment; it was emphasized that only the first roll was to be reported. Subjects were also instructed not to communicate the outcome to anyone else, including the experimenters. The task took approximately 10 min for each group of subjects.

### Data Analysis

#### Genotyping

DNA was extracted either from blood samples using QIAamp DNA Blood Midi Kit (QIAGEN), or from saliva samples collected with Oragene DNA OG-500 tubes (DNA Genotek, Inc., Ottawa, ON, Canada). All subjects’ DNA samples were genotyped with Human Omni Express 12 v1.0 DNA Analysis Kit (Illumina, Inc., San Diego, CA, USA) at the Genome Institute of Singapore.

### Statistical Analysis

To test whether subjects were lying, on average, a non-parametric chi-square test was carried out, comparing the observed distribution to the theoretical uniform distribution expected from a fair die. Based on this, a binomial test was conducted to test the deviation of the frequency of each die outcome from the equal probability predicted. Potential gender differences of lying were also examined using the independent chi-square test.

To investigate the relationship between *TPH2* SNPs and deceptive behavior, a linear regression analysis was conducted, using sex and age as covariates. In order to avoid multiple comparisons, we corrected the *p*-value using the False Discovery Rate (FDR) test (Benjamini et al., 2001). Linkage disequilibrium (LD) structure of the TPH2 gene was plotted in Haploview^3^.

To test the robustness of our results, we further conducted principal components analysis (PCA) and haplotype analysis respectively. We ran PCA over the whole available 29 TPH2 SNPs using an additive model. PCA is regarded as a useful approach to identify the most informative SNPs in genetic analyses for association studies testing multiple SNPs or in correcting for stratification in disease studies (Reich et al., 2008). The first eigenvalue from PCA, which captures a significant fraction of the SNP variation, was used as a single index to represent *TPH2* gene in lying behavior. We then carried out the regression analysis using this eigenvalue as the dependent variable with the reported die roll outcome, similar to the analysis for individual SNPs. Finally, we carried out haplotype analysis in PLINK over those individually significant SNPs of *TPH2* in the previous linear regression at *p* threshold of 0.05.

## Results

### Behavioral Results

**FIGURE 1:**
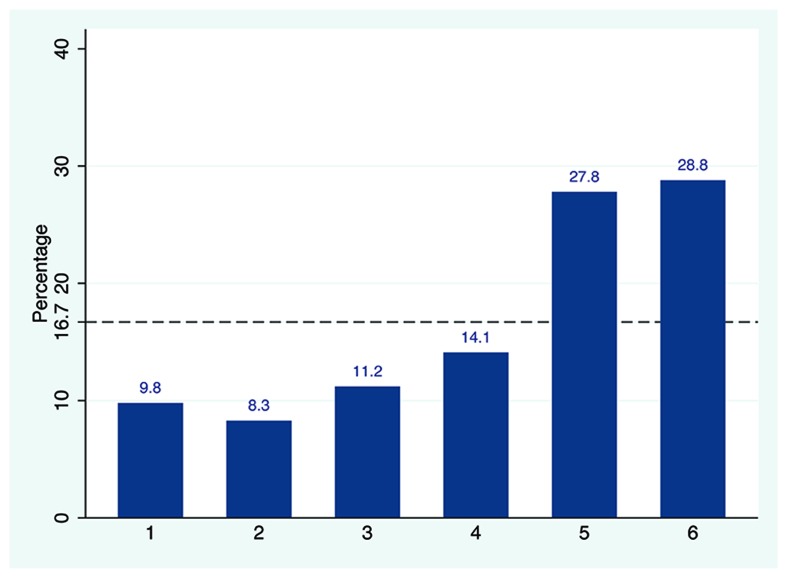
**The observed die-roll outcomes**. The percentage of reported die outcomes for the recruited sample. The dashed line represents the theoretically uniform distribution predicted by chance (16.67% per die side).

As presented in **Figure 1**, the die-roll outcome reported was not equally distributed with average reported number of 4.28 (*SD* = 1.64). A chi-square test of goodness-of-fit confirmed that the reported number was significantly skewed to larger die numbers [χ^2^(5,205) = 52.2, *p* < 0.001], which indicates that some of the subjects were lying. Using the binomial test, we tested whether the frequency reported for each die outcome was significantly different from theoretical 16.7% of fair die. Die outcomes below or equal to 3 (“1,” “2,” and “3”) were significantly under reported as compared to the expected true value of 16.7%, whereas the frequency of “5” and “6” reports were prominently higher than 1/6 (see Supplementary Table S1 for details). As there was no apparent reason to assume that anyone reporting a “1” would be lying, it would be fairly safe to assume that about 58% of subjects were “completely honest.” Intriguingly, “incomplete cheating” was also observed in our data as significantly more than 1/6 of the subjects reported “5” and this showed that some subjects neither reported the truth nor maximized profit by reporting “6.” The proportion of “income-maximizers” was 14.5%.

### Genetic Results

#### Individual SNPs

We first analyzed the correlation between each one of the 29 SNPs and the die-roll outcome using linear regression analysis. For deceptive behavior, 18 of 29 SNPs were significant at *p*-value of 0.1; of these, 15 SNPs were significant at *p* < 0.05. After FDR correction for multiple comparisons, 16 SNPs were significant at *p*-value < 0.1 and eight SNPs reached significance at *p* < 0.05 (see Supplementary Table S2 for details). No effect of gender and age was observed (*p*_age_ = 0.234, *p*_gender_ = 0.789, See Supplementary 1.1, Figure S1 for details). Additionally, both IQ measured by Raven’s progressive matrices and the socioeconomic status (SES) represented by family income had no significant effects (*p*-value equals to 0.28 and 0.35 respectively) on explaining the individual heterogeneity of the reported dice roll. The regression analysis revealed that 16 out of 29 SNPs were significant at *p* < 0.05 after controlling for all demographic information including age, sex, IQ, and SES.

**FIGURE 2:**
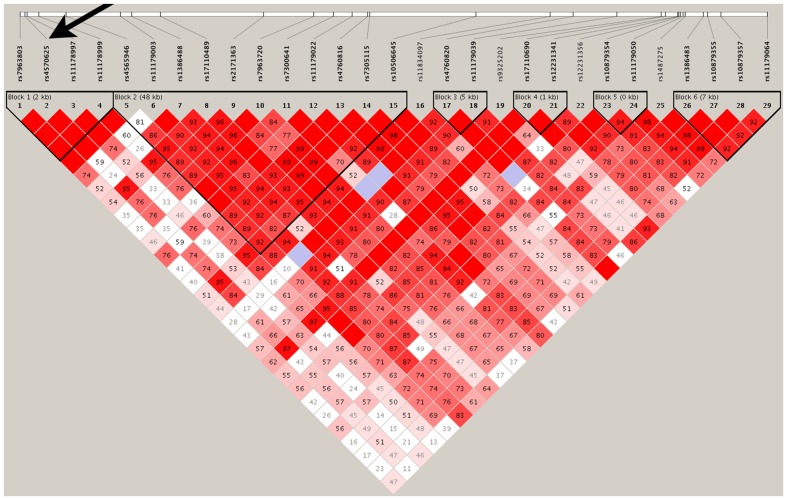
**Linkage disequilibrium: marker-to-marker D’ relation of the 29 examined TPH2 SNPs**. Six haplotype blocks were observed for the total 29 SNPs extracted from the GWAS dataset. SNP rs4570625 was located at the first haplotype block and indexed by an arrow. The LD values were calculated using Haploview (http://www.broad.mit.edu/mpg/haploview/).

As shown in **Figure 2**, except for four SNPs (rs11834097, rs9325202, rs12231341, and rs1487275), the remaining 25 SNPs were in strong LD with each other and comprised six haplotype blocks. We further examine the robustness of the single SNP analysis using two independent strategies: haplotype analysis and PCA.

#### PCA Analysis

Polymerase chain reaction analysis revealed that the first eigenvector extracted from all 29 *TPH2* SNPs had a value of 13.27 and explains approximately 45% of the overall variance (Supplementary Figure S2). We used this first eigenvector as the independent variable as a proxy for genotype in the linear regression with reported die outcome and found a significant correlation with die outcome (*p* = 0.013).

#### Haplotype Analysis Yin–Yang Configuration

Haplotype analysis revealed significant association between the most common (*p* = 0.008, β = 0.505) and second most common (*p* = 0.007, β = −0.516) *TPH2* haplotypes (15 SNPs) in opposite directions (**Table 1**). Furthermore, these two haplotypes (type 2 and type 4) have no overlapping alleles at each and every SNP site, which is consistent with a Yin Yang configuration as reported for a number of other human genes (Zhang et al., 2003) and specifically as we previously reported for *TPH2* (Manor et al., 2008).

**Table 1:** Five haplotype of the 15 *TPH2* SNPs.

Type	Haplotype	Frequency	β	*p*
1	AAAGGAAAAGAAGGG	0.0549	−0.173	0.58
2	**CGGAAGGGGAAAGGG**	0.301	0.525	0.00567
3	AAGAAGGGGAAAGGG	0.0532	0.02	0.957
4	**AAAGGAAAAGGCAAA**	0.206	−0.462	0.0153
5	AAAGGAGGGGGAAAA	0.0894	−0.113	0.674

*Types 2 and 4 (highlighted) are the most two frequent haplotypes and they have no duplicated alleles at each SNP site, consistent with a phenomenon known as Yin and Yang configuration.*

**FIGURE 3:**
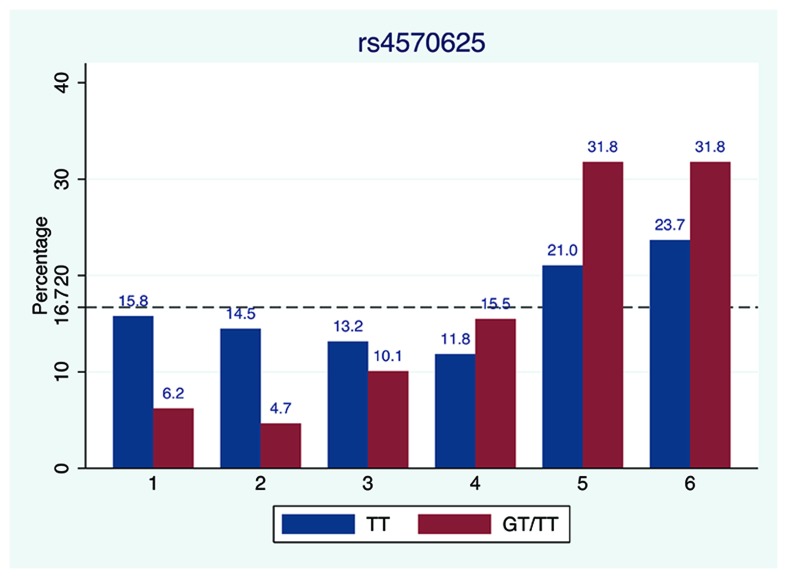
**Die-roll outcome comparison between GG/CG and TT carrier of SNP rs4570625 of ***TPH2*** gene**. The percentage of reported die outcomes stratified by genotype of SNP rs4570625. The dashed line represents the theoretically uniform distribution predicted by chance (16.67% per die side).

We further examined SNP rs4570625 (−703 G/T SNP) in greater detail since several studies of *TPH2* focused on this particular variant (Gao et al., 2012) which is located in the upstream regulatory promoter region 5′-UTR of the *TPH2* gene. As presented in **Figure 3**, the percentage of TT genotype carrier of rs4570625 (15.8%) reporting “1” (truly honest report) was more than twofold greater for carriers of GG genotype (6.2%). Notably, the TT carriers, who reported die number 1, 2, or 3 were not significantly less than 16.7% (*p*_1_ = 1, *p*_2_ = 0.76, and *p*_3_ = 0.54, respectively), strongly suggesting that this genotype group honestly reported the real outcome. In contrast, for each die-outcome (1–6) the GG/TG carriers reported significantly higher (die numbers 5–6) or lower (die numbers 1–3) than 16.7% (*p*_1_ < 0.001, *p*_2_ < 0.001, *p*_3_ = 0.044, *p*_5_ < 0.001, and *p*_6_ < 0.001, respectively). A two-sample Wilcoxon rank-sum (Mann–Whitney) test was administered and a significant difference was observed between TT versus GT/GG genotypes (*Z* = −2.908, *p* = 0.0036). Given our sample size, we further run a permutation test to confirm the robustness of our results and observe a *p*-value of 0.0009. In general, subjects who carry G (TG or GG genotype) allele were inclined to report higher number than those who are AA carriers.

## Discussion

Lying is ubiquitous in daily life from the banking industry (Cohn et al., 2014) to individual cheating on taxes, accepting bribes, skipping on public transit fares, and claiming sick days (Loewen et al., 2013). Moreover, such commonly encountered dishonest behaviors show moderate heritability. In order to further parse the genetic underpinnings of deceptive behavior, we implement a candidate gene approach and examine whether polymorphisms of the serotonin gene *TPH2* can modulate individuals’ inclination to deceive. First, and consistent with previous studies (Mazar et al., 2008; Shalvi et al., 2011; Fischbacher and Heusi, 2013), our behavioral results reveal that individuals do tend to tell lies. We further show a significant association between SNP variants across *TPH2* and the reported die-roll by single SNP analysis, PCA as well as haplotype analysis. To the best of our knowledge, this is the first report of a common polymorphism associated with lying behavior for a small monetary gain carried out in a laboratory setting following the stringent guidelines of behavioral economics.

Subjects’ deceive but do not entirely and in these Han Chinese undergraduates the distribution of the die-roll outcome is remarkably similar to that observed in Caucasians (Mazar et al., 2008; Shalvi et al., 2011; Fischbacher and Heusi, 2013). This cross-cultural identification of deception behavior further suggests that our specific genetic findings might also extend to other ethnic groups. The neurogenetic strategy we implemented in the current report not only identifies a specific gene that is provisionally contributing to lying but importantly also shows the relevancy of serotonergic neural pathways to this behavior. 5-HT is a phylogenetically ancient molecule and serotonergic neural systems have become increasingly complex with more than a dozen serotonin receptors currently known. The role of 5-HT in animal as well as human behavior is complex but overall, 5-HT like other biogenic amines (norepinephrine and dopamine) acts as a neuromodulator. Interestingly, it has been proposed that biogenic amines such as serotonin can spread across large regions of synaptically dense regions (i.e., “volume transmission”; Agnati et al., 2006). Indeed, this aspect of serotonin neurotransmission positions this molecule to have a pervasive influence on behavior. Specifically, it has been suggested that serotonergic brain projections orient behavior toward a drive to withdraw and an inhibition of behavior (Tops et al., 2009).

The results with SNP rs4570625 G/T are especially informative. Subjects with the G allele reported relatively higher number of die-roll, viz., more lying. Interestingly, the G allele is a risk allele in a number of psychiatric disorders. The G allele has been associated with post-stroke anxiety in Han Chinese (Chi et al., 2013), as well as panic disorder in Caucasians (Kim et al., 2009). In a recent meta-analysis, the G allele was associated with major depressive disorder (Gao et al., 2012). At the functional neural level, this upstream regulatory region SNP rs4570625 correlates with functional MRI response of the amygdala (Inoue et al., 2010). Most tellingly, the G allele of rs4570625 was significantly more frequent in children with higher levels of tic symptoms in Chinese subjects diagnosed with Tic Disorder (TD; Zheng et al., 2013). Additionally, the ADHD subjects carrying the G allele in the Go/no go task are characterized by more errors of commission (Baehne et al., 2009). Both the TD and ADHD Go/no go results lead us to conjecture that G allele subjects are more impulsive and characterized by deficits in executive function and self-control (Muraven et al., 2006; Mead et al., 2009), a prerequisite condition for lying behavior.

Considerable evidence suggests that behavioral traits such as impulsivity result in part from a deficit in serotonergic transmission (Lucki, 1998). 5-HT appears to lessen attention to current motivational stimuli and thereby inhibiting behavior by shifting decision-making to considerations of longer-term consequences and delay immediate gratification (Carver and Miller, 2006). Hence, decreased serotonergic tone perhaps due to the presence of the rs4570625G allele would reduce behavioral constraints and lead to impulsive and less considered action as evidenced in the results herein for the die under cup experiment.

Notably, Crockett and her collaborators have pioneered the use of serotonergic agents on moral judgment in human (Crockett et al., 2008, 2010, 2013, 2015; Crockett, 2009; Siegel and Crockett, 2013; Crockett and Fehr, 2014). Overall, these studies indicate that serotonin modulates human attitudes toward harm and fairness. Serotonin impacts harm aversion in moral judgment and aversive evaluations more generally. Such a model suggests the notion that serotonin influences social behavior by shifting social preferences in the positive direction, enhancing the value people place on others’ outcomes. However, it is difficult to relate these mechanisms of serotonin action on moral behavior with the die-under-cup task behavior we observe in our student population and its correlation with *TPH2* polymorphism. In the paradigm implemented in the current report, it is likely that the widespread cheating behavior observed in this task indicates that the subjects see no harm done as a result of their anonymous actions. Additionally, as a task without component of social interactions, fairness plays little role for the judgment to cheat or not to cheat in the die-under-cup paradigm. Altogether, we suggest the effect of the *TPH2* variants we have studied are related to the impulsivity facet of serotonin’s actions (Manor et al., 2002; Zoratto et al., 2013; Zupanc et al., 2013) viz., snap judgments to earn a small amount of money where no harm or unfair treatment is apparent.

In general, our study is driven by a strongly motivated candidate gene hypothesis that variants in *TPH2* contribute to individual differences in honest behavior. Notably, this hypothesis makes eminent biological sense. The importance of the study is its implementation of a neurogenetic strategy to identify which neurotransmitter pathways are contributing to one facet of moral decision-making, viz., to cheat or not to cheat for small monetary reward. Although, any one common genetic polymorphism contributes only incrementally to complex behavior and are therefore not especially helpful in prediction, we believe the perhaps greater value of genetic analysis for psychological traits is to focus attention on which biological pathways underlie these behaviors. The current study succeeds in this aim and identifies serotonin, and the rate-limiting enzyme in its biosynthesis, as a salient mechanism contributing to lying behavior. Finally, we note that as for all candidate gene studies replication is essential to confirm the robustness of the current findings.

## Author Contributions

QS, MT, EW, and RE conceived the project. EW, SC, and RE supervise the project. QS, MT, EW, EH, SC, and RE designed the experiments. QS and MT carried out the experiments. QS, MT, and EH analyzed the data. QS, MT, EW, EH, SC, and RE wrote the manuscript.

## Conflict of Interest Statement

The authors declare that the research was conducted in the absence of any commercial or financial relationships that could be construed as a potential conflict of interest.

## Footnotes

1 http://www.orsee.org

2 http://b2ess.nus.edu.sg/

3 https://www.broadinstitute.org/scientific-community/science/programs/medical-and-population-genetics/haploview/haploview
