# Supplementary material for: To Cheat or Not To Cheat: Tryptophan Hydroxylase 2 SNP Variants Contribute to Dishonest Behavior
Source: Front Behav Neurosci. 2016 May 2;10:82. doi: 10.3389/fnbeh.2016.00082 (PMC4852200; doi:10.3389/fnbeh.2016.00082)
Supplement: Supplementary file 1 [file Presentation_1.PDF]

frontiers

Supplementary Material

To cheat or not to cheat: *Tryptophan hydroxylase 2* SNP variants contribute to dishonest behavior

Qiang Shen*, Teo Meijun, Eyal Winter, Einav Hart, Soo Hong Chew* and Richard P. Ebstein*

Qiang Shen, johnsonzhj@gmail.com , Chew Soo Hong, ecscsh@nus.edu.sg, Richard P. Ebstein, psyrpe@nus.edu.sg

1. Supplementary results

Gender comparison

Chi-square test of independence did not show significant gender effects on the die-roll outcome frequencies (χ^2 (5,205) = 6.79, p = 0.236). Interestingly, as can be seen in Figure S1, more females reported "1" (13.5%) – the "truly honest" report, as compared with males (5.9%). Further, more "incomplete cheating" was observed for females than for males (26.0% versus 23.8%).

2. Supplementary Figures and Tables

2.1 Supplementary Figures

Figure S1. Gender comparison of die-roll outcome.

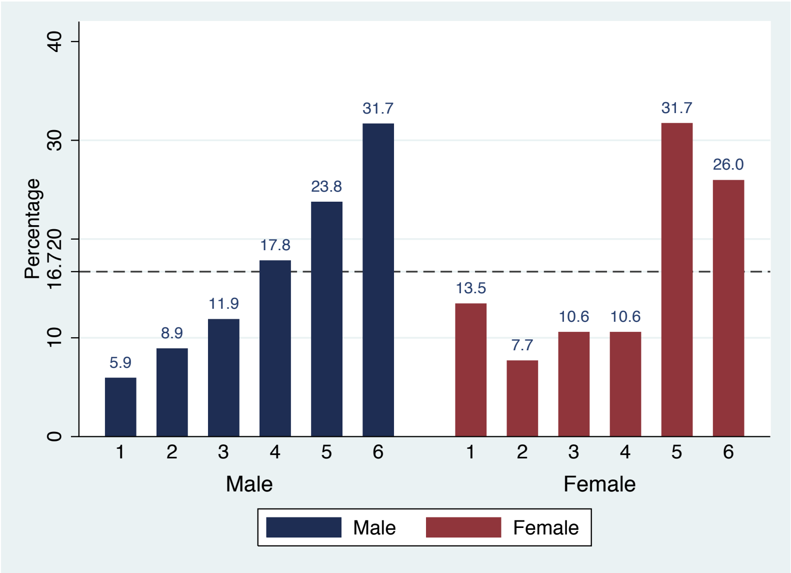

Note. The percentage of reported die outcomes stratified by gender. The dashed line represents the theoretically uniform distribution predicted by chance (16.67% per die side).

Figure S2. Scree plot of principle component analysis.

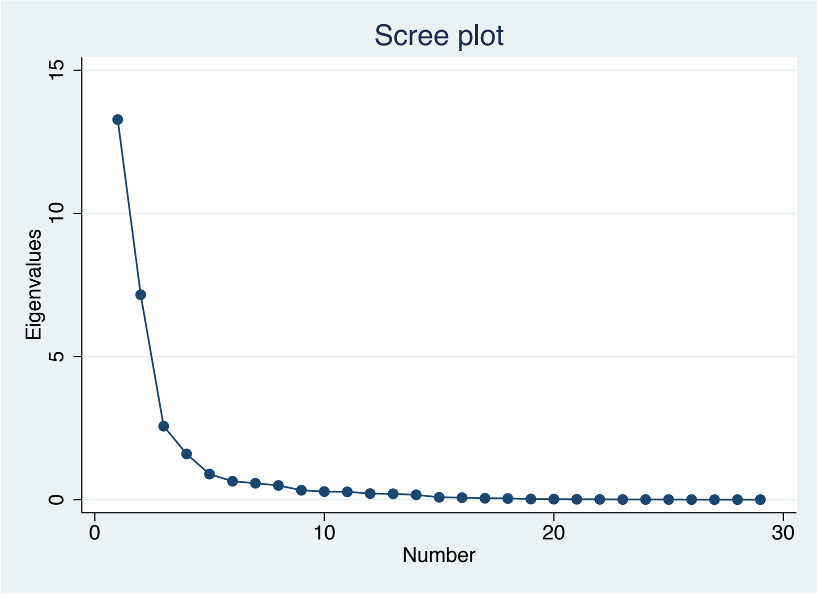

Note. Scree Plot of the PCs from the principal component analysis of 29 available SNPs of TPH2 with the additive model. X-axis represents the PCs and Y-axis indexes the corresponding eigenvalue for each PC.

2.2 Supplementary Tables

Table S1. The summarized results and p value of the binominal test

Die	Subjects	Number of Reports	Percent (%)	Predicted Uniform (%)	p-value
1	205	20	9.76	16.7	0.006
2	205	17	8.29	16.7	0.0007
3	205	23	11.22	16.7	0.039
4	205	29	14.15	16.7	0.399
5	205	57	27.80	16.7	0.00007
6	205	59	28.78	16.7	0.000020

Table S2. Linear regression results with additive model

SNP	Genotype	Subjects	Coefficient	P	P_FDR
rs10506645	A-G	205	-0.3821	0.01766	0.0569
rs10879354	A-G	205	0.4634	0.003808	0.02961
rs10879355	A-G	205	0.4822	0.003251	0.02961
rs10879357	A-G	204	0.417	0.01115	0.04584
rs11178997	A-T	205	0.001718	0.993	0.993
rs11178999	A-G	202	0.4388	0.0073	0.04221
rs11179003	A-G	204	0.1744	0.4592	0.6342
rs11179022	A-G	205	0.1265	0.5737	0.7397
rs11179039	A-G	205	0.2804	0.08865	0.1428
rs11179050	A-G	205	0.4752	0.004085	0.02961
rs11179064	A-G	204	-0.06099	0.7633	0.8913
rs11834097	A-G	205	0.2984	0.06043	0.1031
rs12231341	A-G	205	-0.01961	0.9217	0.9884
rs12231356	A-G	205	-0.1765	0.3917	0.5679
rs1386483	A-G	205	0.4802	0.003567	0.02961
rs1386488	A-C	205	0.1178	0.5867	0.7397
rs1487275	A-C	205	-0.3994	0.01967	0.05705
rs17110489	A-G	205	-0.329	0.05045	0.09144

rs17110690	A-G	205	-0.3403	0.04695	0.09077
rs2171363	A-G	205	0.3547	0.02625	0.06168
rs4565946	A-G	205	0.2506	0.1739	0.2655
rs4570625	A-C	205	0.4307	0.008733	0.04221
rs4760816	A-G	205	0.35	0.02979	0.06171
rs4760820	C-G	202	0.07595	0.7862	0.8913
rs7300641	A-C	205	-0.05057	0.7991	0.8913
rs7305115	A-G	204	0.3579	0.02765	0.06168
rs7963720	A-G	205	0.3547	0.02625	0.06168
rs7963803	A-C	203	0.01124	0.9543	0.9884
rs9325202	A-G	205	-0.3933	0.01265	0.04584
